# *Artemisia* Extract Suppresses NLRP3 and AIM2 Inflammasome Activation by Inhibition of ASC Phosphorylation

**DOI:** 10.1155/2018/6054069

**Published:** 2018-03-04

**Authors:** Su-Bin Kwak, Sushruta Koppula, Eun-Jung In, Xiao Sun, Young-Kyu Kim, Myong-Ki Kim, Kwang-Ho Lee, Tae-Bong Kang

**Affiliations:** ^1^Department of Applied Life Science, Graduate School, Konkuk University, Chungju, Republic of Korea; ^2^Department of Food Science and Engineering, Seowon University, Cheongju, Republic of Korea; ^3^Department of Biotechnology, College of Biomedical & Health Science, Research Institute of Inflammatory Diseases, 268 Chungwon-daero, Chungju, Republic of Korea

## Abstract

*Artemisia princeps* var. *orientalis* (Asteraceae, *A. princeps*) is a well-known traditional medicinal herb used for treating various inflammatory disorders in Korea, Japan, China, and other Asian countries. In the present study, we investigated the effects of *A. princeps* extract (APO) on interleukin- (IL-) 1*β* regulation and inflammasome activation in bone marrow-derived macrophages (BMDMs) and monosodium urate- (MSU-) induced peritonitis mouse model *in vivo*. The APO treatment to BMDMs primed with lipopolysaccharide (LPS) attenuated the NLRP3 and AIM2 inflammasome activation induced by danger signals, such as ATP, nigericin, silica crystals, and poly (dA:dT), respectively. Mechanistic study revealed that APO suppressed the ASC oligomerization and speck formation, which are required for inflammasome activation. APO treatment also reduced the ASC phosphorylation induced by the combination of LPS and a tyrosine phosphatase inhibitor. *In vivo* evaluation revealed that intraperitoneal administration of APO reduced IL-1*β* levels, significantly (*p* < 0.05) and dose dependently, in the MSU-induced peritonitis mouse model. In conclusion, our study is the first to report that the extract of *A. princeps* inhibits inflammasome activation through the modulation of ASC phosphorylation. Therefore, APO might be developed as therapeutic potential in the treatment of inflammasome-mediated inflammatory disorders, such as gouty arthritis.

## 1. Introduction

Interleukin- (IL-) 1*β* is a potent proinflammatory cytokine that mediates various inflammatory responses and is involved in a variety of cellular activities, such as cell differentiation, proliferation, and chemotaxis [1–3]. IL-1*β* is generated from a biologically inactive form (pro-IL-1*β*) in the cytoplasm and is activated through cleavage by caspase-1 in a special protein complex called the inflammasomes [4]. Inflammasomes are innate immune system complexes that regulate the activity of the caspase-1 response to bacterial [5], viral [6], and fungal [7, 8] infections, as well as even sterile stressors [9]. Activation of the inflammasome is therefore ultimately involved in IL-1*β* production and pyroptosis [10, 11], a type of cell death that is dependent on caspase-1 [10] and gasdermin D eliciting inflammation [12]. To date, several types of inflammasomes have been reported, such as NLRP1, NLRP3, and NLRC4, including the intracellular NOD-like receptor family and the absence in melanoma 2 (AIM2) inflammasome [7]. Among them, NLRP3 inflammasome has been most extensively studied. Generally, its activation requires two signals [13]: the first signal is stimulated by pathogen-associated molecular patterns (PAMPs), such as LPS, bacterial RNA, and viral DNA, and leads to NF-*κ*B-dependent expression of pro-IL-1*β* and NLRP3. The second signal is stimulated by damage-associated molecular patterns (DAMPs), such as ATP, pore-forming toxins (e.g., nigericin), crystalline materials, and fatty acids. The NLRP3 inflammasome also consists of an adapter protein apoptosis-associated speck-like protein containing the caspase activation and recruitment domain (CARD) ASC and procaspase-1 [14, 15]. Under appropriate conditions, interactions among these proteins tightly regulate inflammasome function to ensure immune activity.

The NLRP3 inflammasome is also relevant in metabolic diseases, such as gouty arthritis [4] and type 2 diabetes mellitus [16]. Therefore, exploring novel agents that have regulatory activity on the NLRP3 inflammasome might be an ideal therapeutic strategy for the treatment of inflammasome-mediated inflammatory diseases. Enormous efforts have been made in drug discovery, and in particular, traditional medicines have been important resources for treating inflammatory disorders [17, 18].

*Artemisia princeps* var. *orientalis* (*A. princeps*), from the family Asteraceae, is an important medicinal herb with various pharmacological properties including anti-inflammatory effects [19–21]. *A. princeps* is widely used in Korea, Japan, China, and other Asian countries as a traditional adjunct in various food preparations, such as rice cakes and soups, and as a herbal medicine in Korea for inflammation and circulatory diseases, such as dysmenorrhea. Pharmacologically, *A. princeps* possesses antioxidant, anticancer [19, 21], anti-inflammation, and analgesic properties [20]. However, to date, no report exists on *A. princeps* for its possible effects on inflammasome activation. In this study, we investigated the effects of *A. princeps* extract on NLRP3 and AIM2 inflammasome activation and explored the underlying mechanism in bone marrow-derived macrophages (BMDMs) *in vitro* and MSU-induced mouse peritonitis model *in vivo*.

## 2. Materials and Methods

### 2.1. Reagents

RPMI 1640, fetal bovine serum (FBS), Dulbecco's modified Eagle's medium (DMEM), penicillin-streptomycin, and trypsin-EDTA were purchased from Gibco (Grand Island, NY, USA). The ELISA kit for IL-1*β* (DY401) and the IL-1*β* antibody (AF401) were purchased from R&D Systems (Minneapolis, MN, USA), and the ELISA kit for tumour necrosis factor- (TNF-) *α* was obtained from eBioscience (San Diego, CA, USA). Antibodies against NLRP3 (Cryo2), ASC (AL177), and capase-1 (p20) (AG-20B-0042) were purchased from Adipogen (CA, USA). Antibodies against caspase-1 (p10), ASC (N-15), and *β*-actin were obtained from Santa Cruz Biotechnology (CA, USA). Silica crystals (tlrl-sio), nigericin (tlrl-nig), ultrapure flagellin from *Salmonella typhimurium* (tlrl-epstfla), poly(deoxyadenylic-deoxythymidylic) acid (poly dA:dT, tlrl-patn), monosodium urate (MSU) crystal (tlrl-msu), and carbobenzoxy-valyl-alanyl-aspartyl-[O-methyl]-fluoromethylketone (zVAD-FMK, tlrl-vad) were purchased from InvivoGen (San Diego, CA, USA). LPS (*E. coli* 011:B4), sodium orthovanadate, and ATP were purchased from Sigma-Aldrich (St. Louis, MO, USA). Disuccinimidyl suberate (DSS) was purchased Thermo Fisher Scientific (Waltham, MA, USA). Western blot chemiluminescence reagent kit was purchased from Pierce Chemical (Rockford, IL, USA). Nitrocellulose (NC) membranes and phosphortyrosine antibody (05-1050) were purchased from Millipore Corporation (Bedford, MA, USA). Phosphor-ASC (Tyr-144) antibody was obtained from ECM Biosciences (Versailles, KY, USA).

### 2.2. Plant Material

The dried plant material of *A. princeps* (Cat. number 011–068) was generously supplied by the Korea Research Institute of Bioscience and Biotechnology (KRIBB) and was authenticated by Dr. Shin-Ho Kang, a taxonomist at KRIBB, based on its microscopic and macroscopic characteristics. A voucher specimen (KRIBB number 033–021) was deposited at the herbarium of the Plant Extract Bank at KRIBB for future reference. The *A. princeps* leaf was extracted with 80% methanol in a Soxhlet apparatus for 90 min. The resulting methanolic extract was then concentrated under reduced pressure, lyophilized, and stored at 4°C. The lyophilized powder, designated as APO hereafter, was dissolved in dimethyl sulfoxide (DMSO) and stored as stock until use. The final concentration of DMSO used in the study was never greater than 0.1%.

### 2.3. Animals

Male C57BL/6 mice (weighing 22–25 g, seven weeks old) were purchased from Orient Bio Inc. (Seongnam, Korea) and stabilized under standard conditions (humidity 55 ± 5%, temperature 22 ± 2°C, 12/12 h light/dark cycle) with food and water supplied ad libitum. All animal experiments were performed under the guidelines of the Konkuk University Animal Care Committee. The study protocol was reviewed and approved by the Ethics of Animal Experiments Committee of Konkuk University, Republic of Korea (Permit number KU17050).

### 2.4. Cell Culture

Bone marrow cells were collected by flushing the tibias and femurs from C57BL/6 mice with sterile PBS. The collected cells were cultured for 7 days in nontreated surface cell culture dishes (SPL Life Science Co., Gyeonggi-do, Korea) containing RPMI 1640 supplemented with 10% FBS, 1% penicillin-streptomycin, and 30% L929 cell-conditioned medium (LCM) to promote differentiation into bone marrow-derived macrophages (BMDMs). J774A.1 cells were also cultured in RPMI 1640 supplemented with 10% FBS and 1% penicillin-streptomycin. These cells were incubated overnight at 37°C in a 5% CO_2_ atmosphere.

### 2.5. Inflammasome Activation Assay

BMDMs (1.0 × 10^6^ cells/well) were plated in six-well plates and primed with LPS (100 ng/mL) for 3 h. The medium was then replaced with OptiMEM, and the cells were incubated for 30 min with or without APO (50, 100, and 200 *μ*g/mL) or zVAD, before treatment with poly (dA:dT) (2 *μ*g/mL), ATP (5 mM), or nigericin (10 *μ*M) for 1 h or silica crystals (150 *μ*g/mL) or flagellin (1.5 *μ*g/mL) for 3 h.

### 2.6. Cell Viability Assay

BMDMs (5.0 × 10^4^ cells/well) were seeded in 96-well plates and incubated with APO (1.25–200 *μ*g/mL). After incubation for 24 h, cell supernatants were collected by centrifugation at 300 ×g for 5 min and analysed for lactate dehydrogenase (LDH) using an EZ-LDH kit (Dozen, Seoul, Korea).

### 2.7. ASC Translocation and Oligomerization Assay

BMDMs (1.0 × 10^6^ cells/well) were seeded in 6-well plates and lysed with 500 *μ*L of AO buffer (150 mM KCl, 20 mM HEPES-KOH, pH 7.5) containing 1% Triton X-100 and a protease inhibitor cocktail (Roche) for 20 min on ice. The cell lysates were then centrifuged at 3200 ×g for 15 min at 4°C, and the supernatant and pellets were used as the Triton-soluble and Triton-insoluble fractions, respectively. For the ASC oligomerization assay, the Triton-insoluble fraction was washed twice with cold PBS and suspended in 500 *μ*L PBS. The suspended pellets were cross-linked with 2 mM DSS for 30 min and centrifuged at 3200 ×g for 15 min. The pellets were boiled with sodium dodecyl sulfate (SDS) sample buffer for 5 min for Western blot analysis.

### 2.8. Western Blotting

Protein samples were boiled for 5 min at 100°C with sample buffer after determination of the concentration with the bicinchoninic acid (BCA) assay. The samples were resolved via SDS-polyacrylamide gel electrophoresis (SDS-PAGE) and electro transferred onto a nitrocellulose membrane. The membrane was blocked with 5% skim milk in phosphate-buffered saline with Tween 20 (PBST) for 1 h at room temperature and then treated with primary antibodies overnight at 4°C. After washing with PBST, the blot was incubated with HRP-conjugated secondary antibody for 1 h at room temperature. The reactive bands were visualized with ECL Plus (GE Healthcare, Uppsala, Sweden) using a Davinch-Chemi Imaging system (Youngwha Scientific Co., Seoul, Korea).

### 2.9. Immunofluorescence Staining for ASC Specks

BMDMs (3.0 × 10^5^ cells/well) seeded on an eight-well chamber slide were primed and pretreated for 30 min with or without inhibitor or DMSO before treatment with silica crystals (150 *μ*g/mL) for 3 h or poly (dA:dT) (2 *μ*g/mL) for 1 h. The slide was transferred to 4% paraformaldehyde for 20 min on ice and permeabilized with 100% acetone for 10 min at −20°C. The dried slides were then rehydrated with PBS and blocked with 10% horse serum for 1 h. The cells were stained with anti-ASC antibody (N-15) and Cy3-conjugated anti-rabbit antibody. Nuclei were stained with DAPI, and fluorescence microscopy (AX10, Zeiss, Oberkochen, Germany) images were obtained.

### 2.10. Detection of ASC Phosphorylation

The immunoblotting of phosphorylated ASC was performed as described previously [22]. J774A.1 cells (2 × 10^6^ cells/well) or BMDMs (1.5 × 10^6^) were seeded in 6-well plates primed with LPS (37 ng/mL) for 8 h in OptiMEM, then incubated with 1 mM sodium orthovanadate with or without APO for 3 h. Cell lysates were prepared with RIPA buffer (150 mM NaCl, 0.1% SDS, 50 mM Tris, 1.0% NP-40, and 0.5% DOC, pH 8). The phosphorylation of ASC in lysates was detected by standard Western blot assays using the antiphospho-ASC antibody. For ASC precipitation, the lysates were incubated with ASC antibody (N-15) overnight at 4°C, followed by incubation with protein G agarose beads for 4 h at 4°C. The complexes were eluted by boiling with 2× sample buffer and analysed by Western blotting with antiphosphotyrosine antibody.

### 2.11. In Vivo MSU Challenge

For MSU-induced IL-1*β* production in mice, C57BL/6 mice were administered APO (50, 25, and 12.5 mg/kg) and MCC950 (20 mg/kg) intraperitoneally at 12 h and 2 h before injection of MSU (50 mg/kg). The peritoneal lavage samples were obtained 6 h after MSU injection by washing the peritoneal cavity with 1 mL of cold PBS.

### 2.12. Cytokine Analysis by ELISA

The IL-1*β* levels were determined using a mouse IL-1 beta/IL-1F2 DuoSet ELISA kit (R&D Systems, Minneapolis, MN, USA), and TNF-*α* levels were measured using a mouse TNF-*α* ELISA Ready-SET-Go kit (eBioscience, San Diego, CA, USA), according to the manufacturer's instruction. The absorbance measurements were read on a Multiskan GO microplate spectrophotometer (Thermo Fisher Scientific, Waltham, MA, USA).

### 2.13. Fingerprint Analysis of the APO Extract by High-Performance Liquid Chromatography

The APO was dissolved in 80% methanol and centrifuged for 5 min at 3000 rpm for fingerprint analysis. The APO components were determined using a high-performance chromatography (HPLC) system (Shimadzu, Kyoto 211, Japan) equipped with a CBM-20A communications bus module, LC-20AD liquid chromatography pumps, an SPD-M20A diode array detector, an SIL-20AC auto sampler, a DGU-20A3 degasser, and a CTO-20AC column oven. The constituents of the extract were separated on a Shim-pack VP-ODS column (3 × 75 mm, 2.2 mm) at a flow rate of 0.15 mL/min. The injection volume was 10 *μ*L. Molecular weight was determined with an ion trap time-of-flight (IT-TOF) system (Shimadzu, Kyoto, Japan). The mobile phase, consisting of 100% acetonitrile (solvent A) and water containing 0.1% formic acid (solvent B), was run with the gradient program shown in Table 1. The mass range (m/z) was 100–1500 amu.

### 2.14. Statistical Analysis

Data were expressed as the mean ± SEM of at least three independent experiments, each performed in triplicate. Statistical analysis was performed using Student's *t*-test, and *p* values less than 0.05 were considered statistically significant.

## 3. Results

### 3.1. Effect of APO on IL-1*β* Secretion

**Figure 1:**
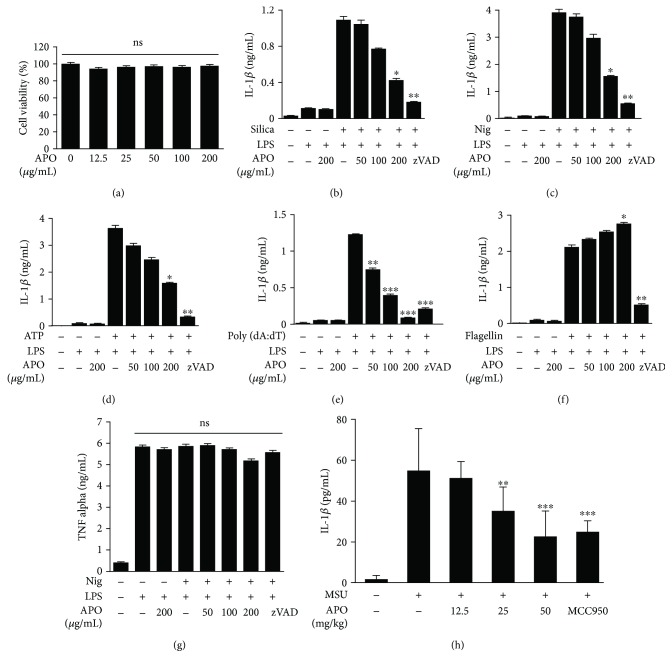
Effect of APO on cell viability and IL-1*β* secretion in LPS-primed BMDMs. (a) BMDMs were treated with the indicated concentration of APO for 24 h. Cell viability was measured by the LDH assay. (b–f) LPS-primed BMDMs were pretreated with APO or zVAD (20 *μ*M), and IL-1*β* secretion was determined by ELISA upon stimulation with (b) 150 *μ*g/mL silica for 3 h, (c) 10 *μ*M nigericin for 1 h, (d) 5 mM ATP for 1 h, (e) 2 *μ*g/mL poly (dA:dT) for 1 h, and (f) 1.5 *μ*g/mL flagellin for 3 h. (g) TNF-*α* release from LPS-primed BMDMs was determined upon stimulation with 10 *μ*M nigericin for 1 h. (h) The impact of APO on MSU-induced IL-1*β* production in mice. Data were expressed as the mean ± SEM (*n* = 3). Statistical analysis was performed using Student's *t*-test. ^∗^*p* < 0.05, ^∗∗^*p* < 0.01, and ^∗∗∗^*p* < 0.001, compared with LPS-primed BMDMs plus respective stimuli. NS, nonsignificant; LPS, lipopolysaccharide; BMDMs, bone marrow-derived macrophages; APO, *A. princeps* extract; TNF, tumor necrosis factor; IL, interleukin; Nig, nigericin; MSU, monosodium urate crystal.

In preliminary experiments, the effect of APO on the viability of BMDMs was evaluated by LDH assay. As shown in Figure 1(a), treatment with APO alone at up to 200 *μ*g/mL did not result in any signs of cytotoxicity, indicating that the concentrations of APO used in this study were nontoxic. We investigated the possible inhibition of NLRP3 inflammasome activation by treating LPS-primed BMDMs with APO 30 min before treatment with various inducers, such as silica, nigericin, and ATP. As shown in Figures 1(b)–1(d), the IL-1*β* secretions were significantly increased (*p* < 0.05) in the LPS-primed BMDMs activated by silica, nigericin, and ATP and these increases were dose dependently inhibited by APO.

Further, we also evaluated whether APO could inhibit the activation of other inflammasomes, such as AIM2 and NLRC4. In agreement with the data shown in Figures 1(b)–1(d), the release of IL-1*β* induced by poly (dA:dT) was significantly (*p* < 0.001) inhibited by APO (Figure 1(e)). However, APO had no influence on flagellin-induced IL-1*β* release (Figure 1(f)). We clarified whether APO specifically inhibited IL-1*β* release by examining the release of another proinflammatory cytokine, TNF-*α*, in the same culture supernatants. As shown in Figure 1(g), APO had no inhibitory effect on TNF-*α* release.

We then investigated the activity of APO on IL-1*β* release *in vivo* in the MSU-induced peritonitis mouse model. The injection of MSU-induced IL-1*β* release in the peritoneal cavities of the mice and the administration of APO or an NLRP3 inflammasome inhibitor, MCC950, significantly reduced this IL-1*β* secretion in a dose-dependent manner (Figure 1(h)).

### 3.2. Effect of APO on Inflammasome Activation and on the Expression of Inflammasome Components

**Figure 2:**
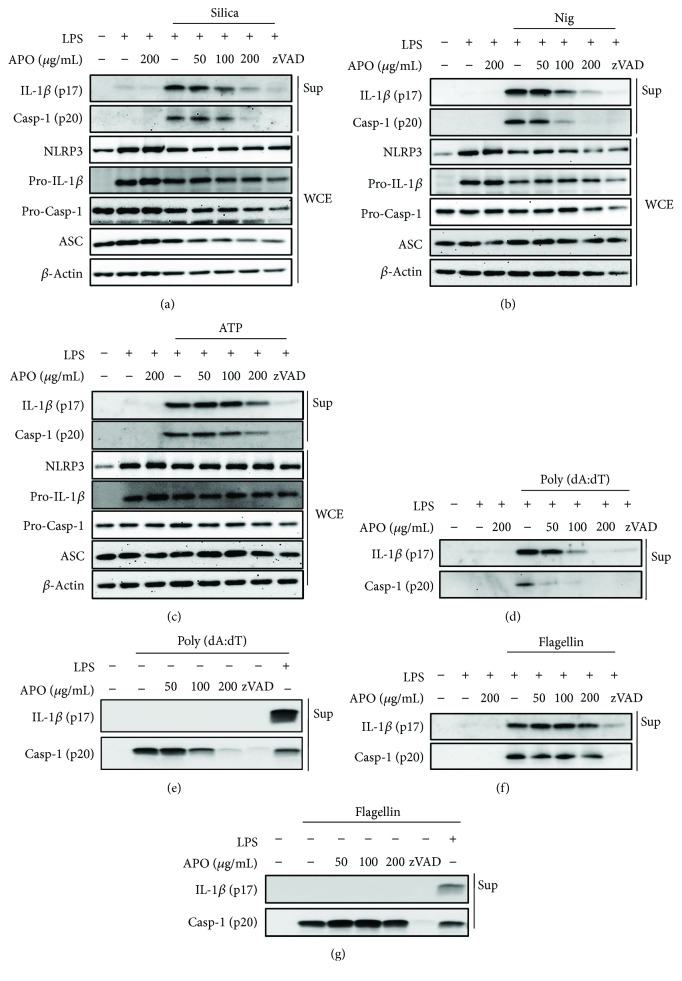
Effect of APO on inflammasome components. LPS-primed BMDMs (a–d, f) and nonprimed BMDMs (e, g) were pretreated with APO or zVAD (20 *μ*M) and stimulated with (a) 150 *μ*g/mL silica for 3 h, (b) 10 *μ*M nigericin for 1 h, (c) 5 mM ATP for 1 h, (d-e) 2 *μ*g/mL poly (dA:dT) for 1 h, and (f-g) 1.5 *μ*g/mL flagellin for 3 h. Mature IL-1*β* and caspase-1 cleavage were measured in supernatant (Sup). NLRP3, pro-IL-1*β*, procaspase-1, and ASC were measured in whole cell extract (WCE) by immunoblotting. *β*-Actin was used as an internal control. LPS, lipopolysaccharide; BMDMs, bone marrow-derived macrophages; APO, *A. princeps* extract; IL, interleukin; Nig, nigericin.

We verified that the inhibitory effect of APO on IL-1*β* release was attributable to the suppression of caspase-1 activation by examining the cleavage of caspase-1 and its substrate, IL-1*β*, by immunoblotting of the supernatant. As shown in Figures 2(a)–2(d), the cleavage of both caspase-1 and IL-1*β* induced by silica, nigericin, and ATP was significantly reduced by APO. However, APO did not affect caspase-1 cleavage by the flagellin-induced NLRC4 inflammasome activation (Figure 2(f)). Because poly (dA:dT)- and flagellin-induced inflammasome activation can occur without a priming signal [23], the impact of APO on inflammasome activation in unprimed BMDMs was examined. Similarly, APO suppressed the cleavage and secretion of caspase-1 from nonprimed macrophages (Figures 2(e) and 2(g)). However, the release of IL-1*β* was not detectable in unprimed BMDMs, which might be due to the lack of pro-IL-1*β* production which is upregulated by the priming signal [24].

Since inflammasome is composed of multiple proteins and its expression affects its activity, we assessed the expression of inflammasome components in the cell lysates. Unlike the cleavage of caspase-1 or IL-1*β* in the supernatants, the expression of NLRP3, ASC, pro-IL-1*β*, and procaspase-1 in the cell lysates was not affected by APO treatment (Figures 2(a)–2(c)).

### 3.3. Effect of APO on the Redistribution, Oligomerization, and Speck Formation of ASC

**Figure 3:**
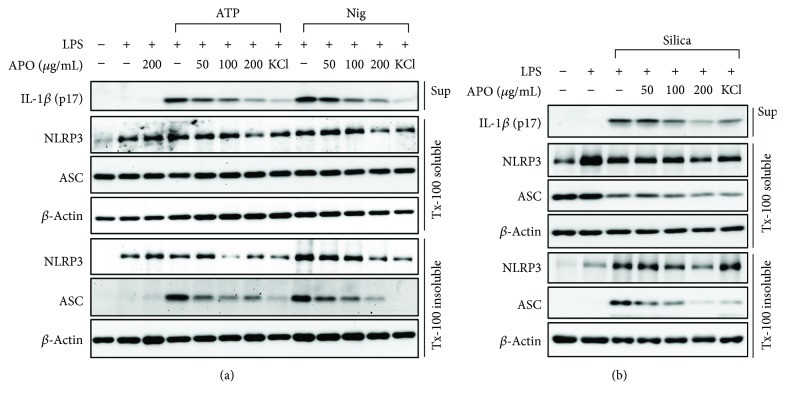
Effect of APO on ASC translocation during inflammasome activation. LPS-primed BMDMs were pretreated with APO or KCl (150 mM), and IL-1*β* secretion was determined by Western blotting upon stimulation with (a) 5 mM ATP, 10 *μ*M nigericin for 1 h, and (b) 150 *μ*g/mL silica for 3 h in the supernatant (Sup). NLRP3 and ASC distribution was estimated by Western blotting of the soluble and insoluble fractions of Triton X-100. *β*-Actin was used as an internal control in each fraction. LPS, lipopolysaccharide; BMDMs, bone marrow-derived macrophages; APO, *A. princeps* extract; IL, interleukin; Nig, nigericin.

During inflammasome activation, ASC translocates to the detergent-insoluble fraction and forms ASC specks due to the aggregation of ASC oligomers [25]. Therefore, we examined the impact of APO on the translocation and speck formation of ASC in BMDMs. It has been reported that K^+^ efflux plays an essential role in NLRP3 activation and the elevation of extracellular potassium concentration inhibits inflammasome activation by various NLRP3 activators [26, 27]. Thereby, some BMDMs were stimulated in the presence of 150 mM extracellular KCl as a positive control to inhibit NLRP3 inflammasome activation. As expected, the amount of ASC in the Triton X-100-insoluble fraction greatly increased in LPS-primed BMDMs after stimulation with various NLRP3 inflammasome agonists and it was inhibited by the addition of extracellular KCl. Furthermore, the translocation of ASC was also significantly decreased in a concentration-dependent manner by pretreatment with APO (Figures 3(a) and 3(b)).

**Figure 4:**
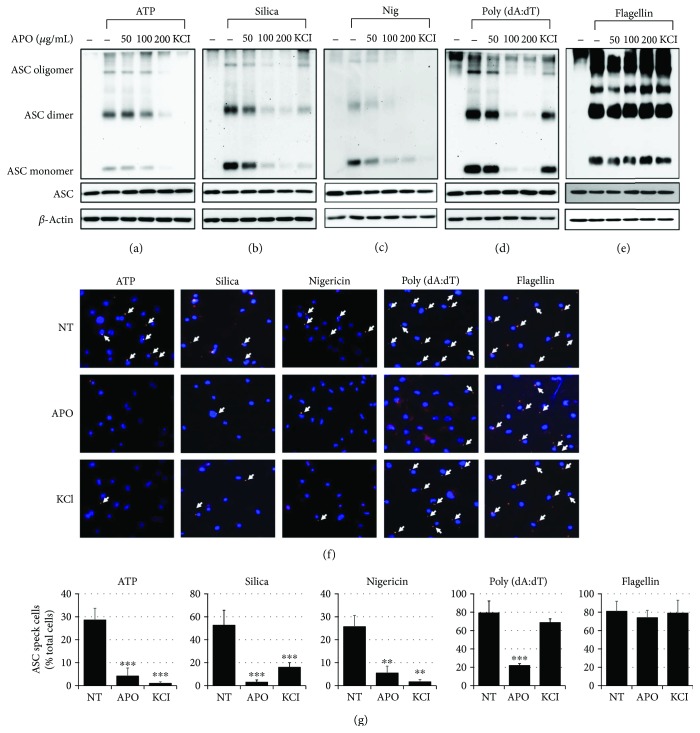
Inhibitory effect of APO on ASC oligomerization. LPS-primed BMDMs were pretreated with APO or KCl (150 mM), and ASC oligomerization in the cells was determined by Western blotting upon stimulation with (a) 5 mM ATP for 1 h, (b) 150 *μ*g/mL silica for 3 h, (c) 10 *μ*M nigericin for 1 h, (d) 2 *μ*g/mL poly (dA:dT) for 1 h, and (e) 1.5 *μ*g/mL flagellin for 3 h. *β*-Actin is used as an internal control. (f) LPS-primed BMDMs were stimulated with 5 mM ATP for 1 h, 150 *μ*g/mL silica for 3 h, 10 *μ*M nigericin for 1 h, 2 *μ*g/mL poly (dA:dT) for 1 h, and 1.5 *μ*g/mL flagellin for 3 h and stained with anti-ASC antibody for ASC specks (red) and by DAPI for nuclei (blue). ASC specks were marked with arrows, and (g) cells with ASC specks are presented as a percentage of the positive cells. Data were expressed as the mean ± SEM (*n* = 3). Statistical analysis was performed using Student's *t*-test. ^∗^*p* < 0.01 and ^∗∗^*p* < 0.001, compared with respective stimuli in LPS-primed BMDMs plus respective inhibitors. APO, *A. princeps* extract; BMDMs, bone marrow-derived macrophages.

We then investigated the possible effects of APO on the oligomerization and speck formation of ASC, which are regarded as hallmarks of inflammasome formation. As shown in Figure 4, APO and extracellular KCl inhibited the ASC oligomerization induced by ATP, silica, or nigericin (Figures 4(a)–4(c)). Similarly, APO also reduced ASC oligomerization in the AIM2 inflammasome activated by poly (dA:dT) (Figure 4(d)), but it did not affect flagellin-induced ASC oligomerization (Figure 4(e)). Furthermore, microscopic analysis showed that APO significantly decreased ASC speck formation resulting from ATP-, silica-, or nigericin-induced NLRP3 inflammasomes and poly (dA:dT)-induced AIM2 inflammasome activation (Figures 4(f) and 4(g)). These results indicate that APO has the potential to inhibit inflammasome activation through regulation of ASC oligomerization and ASC speck formation.

### 3.4. Effect of APO on ASC Phosphorylation

The phosphorylation of ASC appears critical for ASC oligomerization in NLRP3 and AIM2 inflammasomes [28]. Therefore, we examined whether the inhibitory effect of APO on ASC oligomerization was attributable to the inhibition of ASC phosphorylation.

**Figure 5:**
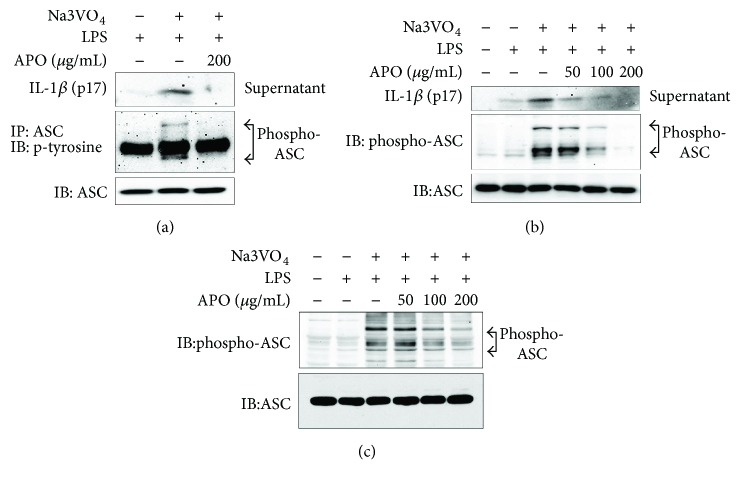
Inhibitory effect of APO on ASC phosphorylation. J774A.1 cells or BMDMs were stimulated with LPS (37 ng/mL) for 8 h and treated with sodium orthovanadate (1 mM) for 3 h. The ASC was enriched by immunoprecipitation. (a) Immunoblot analysis of phosphor-ASC in immunoprecipitates using antibody to phosphorylated tyrosine. ASC was blotted to validate the efficiency of the immunoprecipitation. (b-c) Immunoblot analysis of phospho-ASC in whole cell lysates of (b) J774A.1 or (c) BMDMs using antibody against phospho-specific ASC. IL-1*β* release in cell culture medium was also determined to confirm inflammasome activation. LPS, lipopolysaccharide; APO, *A. princeps* extract; Na_3_VO_4_, sodium orthovanadate.

The inhibition of tyrosine phosphatase greatly enhances the LPS-induced phosphorylation of ASC and facilitates retention of the phosphorylated ACS within mouse macrophage cell line (J774) [22]. Here, we examined the effect of APO on ASC phosphorylation in the J774 macrophage cells treated with a combination of LPS and a potent tyrosine phosphatase inhibitor, sodium orthovanadate. ASC was immunoprecipitated from cell lysates using anti-ASC antibody, and its phospho form was detected by Western blot using antiphosphotyrosine antibody. As anticipated, phosphorylated ASC was detected in the cells treated with sodium orthovanadate, but this phosphorylation of ASC was inhibited by treatment with APO (Figure 5(a)).

Phosphorylation at tyrosine 144 (Y144) of ASC is also viewed as important for inflammasome activation [29]. Therefore, we tested whether the phosphorylation shown in Figure 5(a) occurred at Y144 on ASC by performing Western blot analysis of the total cell lysates of J774 and BMDMs using an antibody specific for phosphorylated ASC on Y144. In agreement with Figure 5(a), the phospho form of ASC was detected in both J774 and BMDM cells treated with LPS and with sodium orthovanadate, but APO dose dependently inhibited this ASC phosphorylation (Figures 5(a) and 5(c)).

### 3.5. HPLC Fingerprint Analysis of APO

**Figure 6:**
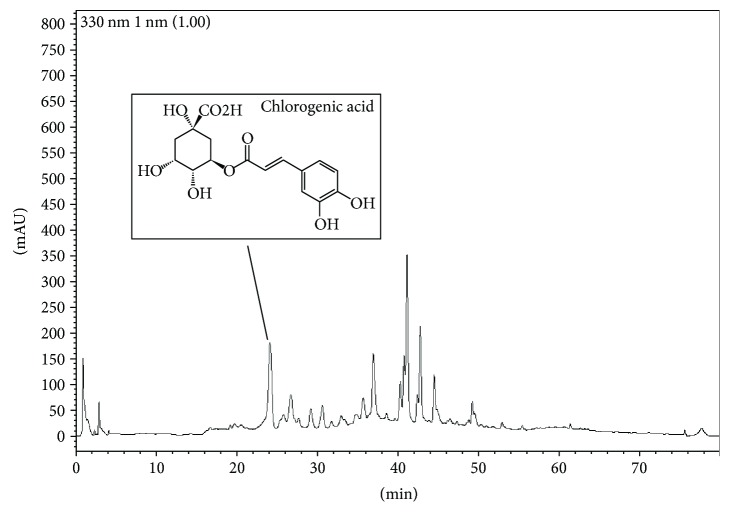
High-performance liquid chromatography (HPLC) fingerprint analysis of APO. The APO components in 100% methanol were determined through an HPLC system for fingerprint analysis as mentioned in Section 2.14. APO: *A. princeps* extract.

The HPLC fingerprint analysis of APO revealed several characteristic peaks at identifiable intensities (Figure 6). Previous reports showed that APO contains several phenolic compounds, such as caffeoylquinic acids, chlorogenic acid, and neochlorogenic acid [30].

Although we could not analyse all the obtained peaks for their respective constituents, we found chlorogenic acid to be one of the major constituents in the extract used in this study.

## 4. Discussion

The activation of inflammasomes usually occurs in response to various pathogenic and physiological stimuli and induces the secretion of proinflammatory cytokines, such as IL-1*β* and IL-18, and the proinflammatory death process called pyroptosis [10, 11]. Therefore, inflammasomes are important components of the innate immune system and play an essential role in the clearance of pathogens and damaged cells [10–12]. However, dysregulation of inflammasomes leads to a broad spectrum of inflammatory diseases, including gouty arthritis [4]. Therefore, targeting inflammasomes could be an effective and ideal strategy for controlling inflammasome-mediated diseases.

*Artemisia* species have been extensively used in traditional Asian medicine and have been noted for their anti-inflammatory properties [20, 28]. In the present study, we investigated the effect of APO on the activation of inflammasomes from various perspectives, and we observed that APO significantly inhibited activation of the NLRP3 and AIM2 inflammasomes but not the activation of NLRC4 inflammasomes. The functional output of inflammasome activation is IL-1*β*, a proinflammatory cytokine that is essential for the amplification of inflammation. IL-1*β* is generated in a proform and is activated and released by caspase-1 during inflammasome activation. Several sensor molecules have thus far been identified in inflammasomes including NLRP1, NPRP3, NLRC4, and AIM2 [7]. Among the different inflammasomes, the NLRP3 inflammasome has been most studied because of its relevance to many metabolic diseases in humans [31].

In the present study, we explored the potency of APO in attenuating the release of IL-1*β* and activating caspase-1 in primary murine macrophages following stimulation with various inflammasome agonists. Interestingly, APO was able to inhibit the IL-1*β* release and caspase-1 response associated with NLRP3- and AIM2-mediated inflammasome activation but not that associated with NLRC4-mediated inflammasome activation. However, APO had no effect on the release of TNF-*α* or the expression of pro-IL-1*β*, procaspase-1, NLRP3, or ASC, which indicates that APO specifically inhibits inflammasome activation.

The activation of inflammasomes can be regulated at both the level of the inflammasome sensor and downstream molecules such as ASC and caspase-1.

Many ASC molecular events associated with inflammasome activation have been reported [25, 29, 32]. During inflammasome activation, the cytosolic macromolecular aggregates formed by ASC oligomerization appear as speck-like aggregates that are resistant to Triton X-100 [29]. Earlier reports have indicated that ASC is translocated from the soluble to the insoluble fraction of Triton X-100 upon inflammasome activation [33]. In light of such reports, in our present study, we examined the redistribution, oligomerization, and speck formation of ASC. We found that APO significantly attenuated NLRP3 and AIM2 agonist-induced ASC speck formation in a concentration-dependent manner. The inhibition of ASC recruitment and oligomerization to the NLRP3 inflammasome complexes has been previously described as a mode of action of certain inhibitors of IL-1*β* secretion, including cytokine-release inhibitory drugs [34–36]. In agreement, our results indicate that APO has the potential to inhibit inflammasome activation through regulation of ASC oligomerization and speck formation.

The specific inhibition of APO on NLRP3- and AIM2-mediated but not on NLRC4-mediated inflammasome activation implied that phosphorylation of ASC might be a possible molecular target of APO. ASC undergoes phosphorylation upon stimulation, which is critical for speck formation and caspase-1 cleavage during inflammasome activation by NLRP3, or AIM2, but not activation by NLRC4 [29, 37]. In the present study, we found that APO disrupted the phosphorylation of ASC induced by a phosphatase inhibitor, sodium orthovanadate [21], in LPS-primed murine macrophage cells. Overall, the present data indicated that APO potently inhibited NLRP3 and AIM2 inflammasome activation *in vitro* in BMDMs and this inhibitory effect of APO might be attributable to its ability to suppress ASC phosphorylation and oligomerization.

The formation of MSU crystals after the release of uric acid is considered as a danger signal in dying cells, seen in the case of gout [38]. The MSU crystals, while being digested by macrophages, cause a parallel stimulation of the NLRP3 inflammasomes, thereby triggering the excessive release of IL-1*β* and leading to the pathogenesis of gout [4]. In the present study, the *in vivo* data agreed with our *in vitro* results that APO inhibited the release of IL-1*β*. Attenuating the release of the inflammatory mediator, IL-1*β* in MSU-induced peritonitis mouse model could represent a potentially effective treatment by APO for gout-related disorders.

Previous reports have indicated that *Artemisia* species contain several active ingredients, including coumarins, glycosides, terpenoids, flavonoids, sterols, and polyacetylenes [39–41]. Some of these compounds are well documented to possess antimalarial, antiviral, antioxidant, and anticancer effects [42]. Compounds such as eupatilin and jaceosidin found in *Artemisia* species have also been reported to show inhibitory effects against IgE-induced hypersensitivity and carrageenan-induced inflammation [43, 44]. Phenolic compounds, such as caffeoylquinic acids, chlorogenic acid, and neochlorogenic acid, which are also present in *Artemisia* species, are also reported to alleviate the oxidative stress and enhance the *in vitro* viability of certain neuronal cells [30]. In the present study, although we identified chlorogenic acid as one of the major peaks detected by ultraviolet absorption, this compound along with other active constituents present in APO might be responsible in exhibiting profound anti-inflammasome activity. However, further work in the isolation of each active constituent present in APO and evaluating their intrinsic mechanisms in the regulation of inflammasome activation is quite essential.

## 5. Conclusions

This report is the first to provide scientific evidence that the extract obtained from *A. princeps* (APO) can serve as a potential regulator of the inflammatory responses through the inhibition of the NLRP3 and AIM2 inflammasome activation. This inhibition was mediated by regulation of ASC oligomerization and phosphorylation. APO also has the potential to ameliorate gouty arthritis via attenuation of inflammasome activation. In conclusion, our present data indicated that APO might be developed as a potential agent for the therapeutic modulation of inflammasome-mediated inflammatory disorders.

## Figures and Tables

**Table 1:** Mobile phase program for HPLC separation.

Time (min)	A% (acetonitrile)	B% (0.1% formic acid)
0	5	95
5	5	95
20	15	85
30	20	80
50	40	60
65	90	10
70	90	10
71	5	95
80	5	95
